# Predicting the outcomes of organic reactions via machine learning: are current descriptors sufficient?

**DOI:** 10.1038/s41598-017-02303-0

**Published:** 2017-06-15

**Authors:** G. Skoraczyński, P. Dittwald, B. Miasojedow, S. Szymkuć, E. P. Gajewska, B. A. Grzybowski, A. Gambin

**Affiliations:** 1 Faculty of Mathematics, Informatics, and Mechanics, University of Warsaw, 02-097 Warsaw, Poland; 2 DARPA Make-It Program & the Institute of Organic Chemistry, Polish Academy of Sciences, Warsaw, Poland; 3 Center for Soft and Living Matter of Korea’s Institute for Basic Science (IBS), Department of Chemistry, Ulsan National Institute of Science and Technology, Ulsan, South Korea

## Abstract

As machine learning/artificial intelligence algorithms are defeating chess masters and, most recently, GO champions, there is interest – and hope – that they will prove equally useful in assisting chemists in predicting outcomes of organic reactions. This paper demonstrates, however, that the applicability of machine learning to the problems of chemical reactivity over diverse types of chemistries remains limited – in particular, with the currently available chemical descriptors, fundamental mathematical theorems impose upper bounds on the accuracy with which raction yields and times can be predicted. Improving the performance of machine-learning methods calls for the development of fundamentally new chemical descriptors.

## Introduction

With the dawn of the big-data era^1–4^, high hopes have been pinned at the ability of machine learning, ML, algorithms^5^ to analyze the large body of existing chemical data, and to derive from it models predictive of various aspects of chemical reactivity. ML methods have already proven very successful in applications ranging from speech or image recognition^6^, to medical diagnostics^7^, bioinformatics^8^, and economics^9^. There have also been some encouraging examples of using ML to predict biological activities of small molecules^10–12^, solubilities^13^, crystal structures^14^, properties of organic photovoltaics^15^ and, recently, compositions of reaction mixtures and/or reaction conditions leading to templated vanadium selenites^16^. This last example is quite spectacular in that machine-learning performed better than the collective knowledge and intuition of chemists who had previously worked on the problem. On the other hand, demonstrations in organic synthetic chemistry are few in number and limited to narrow datasets of similar and/or very simple reaction classes^17–21^. What is largely missing are studies that would quantify the general applicability of ML methods to diverse chemistries.

**Figure 1:**
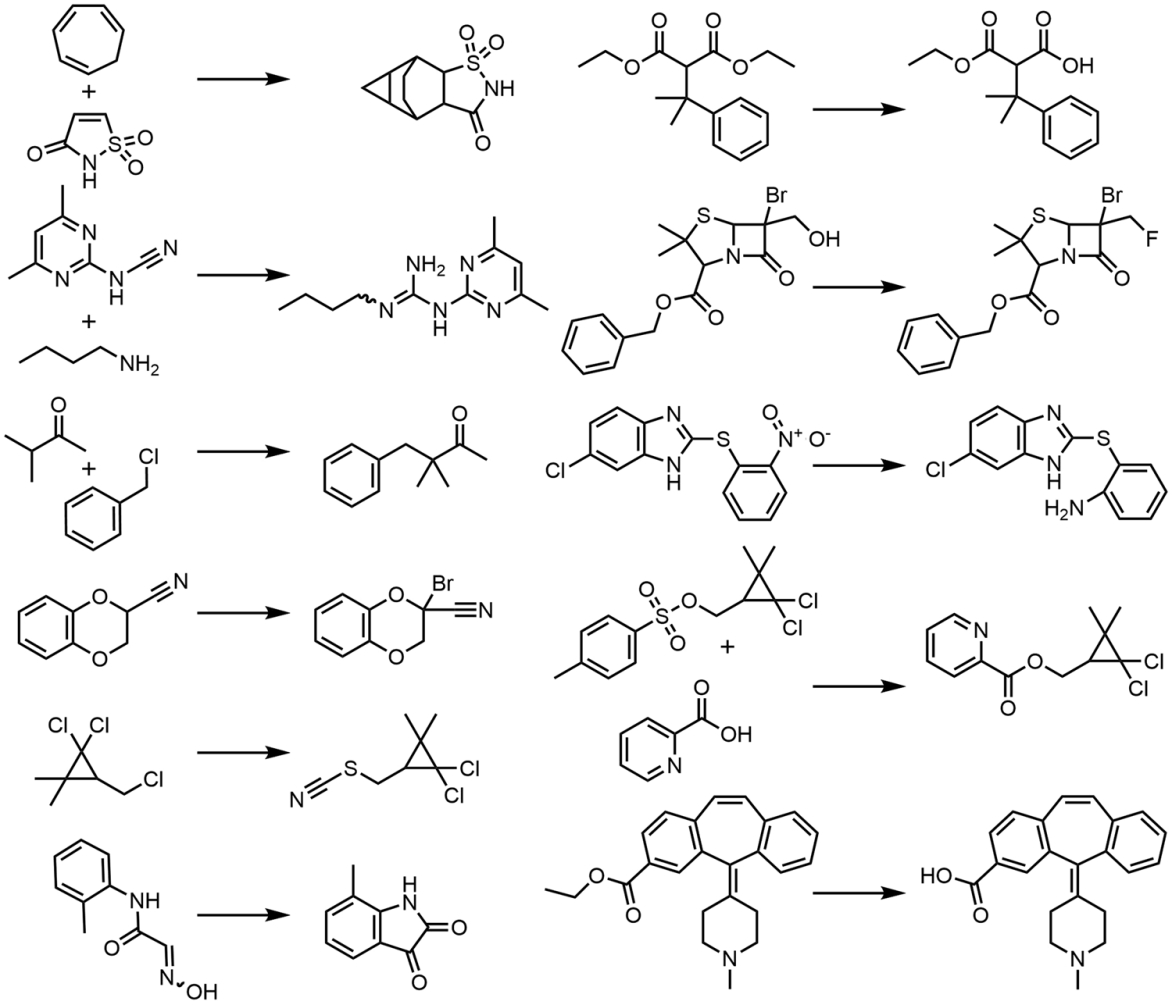
Illustrative reactions from the training set. A small sample of eleven reactions chosen at random from the set of 450,000 reactions analyzed by machine learning methods. The reactions are diverse and span different types of chemistries. Shown here are: cycloaddition, synthesis of guanidines, alkylation of ketones, alpha-bromination of nitriles, substitution of primary alkyl chlorides with thiocyanate anion via S_N_2, synthesis of isatins, ester hydrolysis (in two reactions), fluorination of primary alcohols, reduction of nitro compounds, and esterification of carboxylic acids).

The main objective of this work is therefore to assess in a quantitative manner whether ML methods can predict the outcomes of diverse organic reaction with practically-relevant accuracy. In particular, we use a wide range of currently available chemical descriptors and various ML algorithms to examine whether they can predictively categorize two quantities which are important in organic-synthetic practice and for which ample training examples are available (here, close to 0.5 million reactions each): (i) reaction yields (binary classification high vs. low) and (ii) reaction times (binary classification rapid vs. slow). It is important to note that the training set we use comprises reactions not necessarily accounting for full stoichiometry (i.e., no atomically balanced; see examples in Fig. 1). For reactions with manually curated full stoichiometry, thermodynamic models have recently been shown^22^ to achieve ± 15% accuracy of yield prediction, However, organic reactions are typically drawn by chemists without accounting for all small reagents or side-products – in this light, the current work is a real-world test for the machine learning methods to extract reactivity trends from reactions as they are deposited in the chemical literature or in reaction databases.

The results of our work are somewhat negative but, we believe, thought-provoking. Irrespective of the specific ML method applied, the number of molecules in the training set, or the nature and the number of features/descriptors used to train the model, the accuracy of binary yield prediction is only c.a. 65 ± 5% (i.e., error ~35%) and that of reaction-time prediction, c.a. 75 ± 5% (error ~25%). Another important conclusion of this work is that it can be proven rigorously – by the so-called Bayes classifier error estimates – that with the currently available representations of molecules, (i.e., chemical descriptors), these outcomes cannot be significantly improved. Naturally, it can always be argued that “better” representations of molecules can be developed, though it is somewhat unclear how to account for the immense structural and mechanistic diversity of organic reactions^23^, their often-encountered sensitivity to reaction conditions, or even inherent day-to-day irreproducibilities in reaction outcomes. We will touch upon these interesting issues in the last part of the paper. In the meantime, we see the main virtue of our work in potentially stimulating new research on molecular representations and their use in chemical machine-learning^24^.

## Methods

### Datasets

The initial datasets, courtesy of GSI and Reaxys, comprised ~1,000,000 reactions for which the yields were reported and ~600,000 reactions reporting reaction times. These sets were pruned for incomplete entries and duplicate reactions. When the same reaction was reported with multiple yields, the highest value was taken; if the same reaction was reported with multiple times, the shortest one was chosen. Ultimately, each set comprised ~450,000 reaction entries of which 325,000 had both the yields and times reported (within this common subset, the values of yields and times had a nearly zero correlation, see Supplementary Information, SI, Figure S5).

### ML methods

Various ML methods were implemented and tested including logistic regression^25^, support vector machines (SVM)^26^, neural networks^27, 28^, extremely randomized trees (ERT)^29^ and random forests (RF)^30, 31^. Of these, RF and ERT gave the best – and similar – results (i.e., highest accuracy of classification). For clarity and consistency, RF is described in detail in the discussion that follows (for the results obtained with other methods, see SI).

### Descriptors and fingerprints

In most calculations, two distinct and commonly accepted types of features were used to train the models: (1) Molecular descriptors, summarized in RDKit^32^ and capturing various characteristics of individual molecules (from molecular weight, to the numbers of specific atoms, rings and structural motifs, to various topological indices, etc.; see list at the end of the SI) along with experimental parameters such as solvents and temperature. Models up to almost 400 RDKit descriptors (~200 for substrates and ~200 for products) were constructed and tested. (2) Reaction fingerprints reflecting changes in the molecular features over the reaction process and calculated for each reaction by subtracting the sum of products’ fingerprints from the sum of the reactants’ fingerprints. The fingerprint vectors accounted for 800,000 binary features but were typically very sparse with only several dozen non-zero entries – accordingly, following the procedures from^17^, we condensed them into shorter vectors by hashing bit indices and summing colliding items (though the collisions were very infrequent, meaning that any information loss during compression was negligible). This protocol gave 256-length AP3 fingerprints (Atom-Pairs with maximum path length of three^33^).

### Chemical-linguistic descriptors

In addition, we used descriptors that corresponded to the maximum common substructures between organic molecules. As we showed in ref. 34, the frequency of occurrence of these substructures in large collections of molecules followed the same power-law trend as the frequency of word fragments in English texts – hence, we refer to them as chemical-linguistic descriptors, CLDs. Importantly, such descriptors are more informative than isolated functional groups as they represent characteristic motifs encountered in organic molecules. In the present work, we used up to several thousands of CLDs, alone or in combination with descriptors from the RDKit collection.

### Decision trees and Random Forests

**Figure 2:**
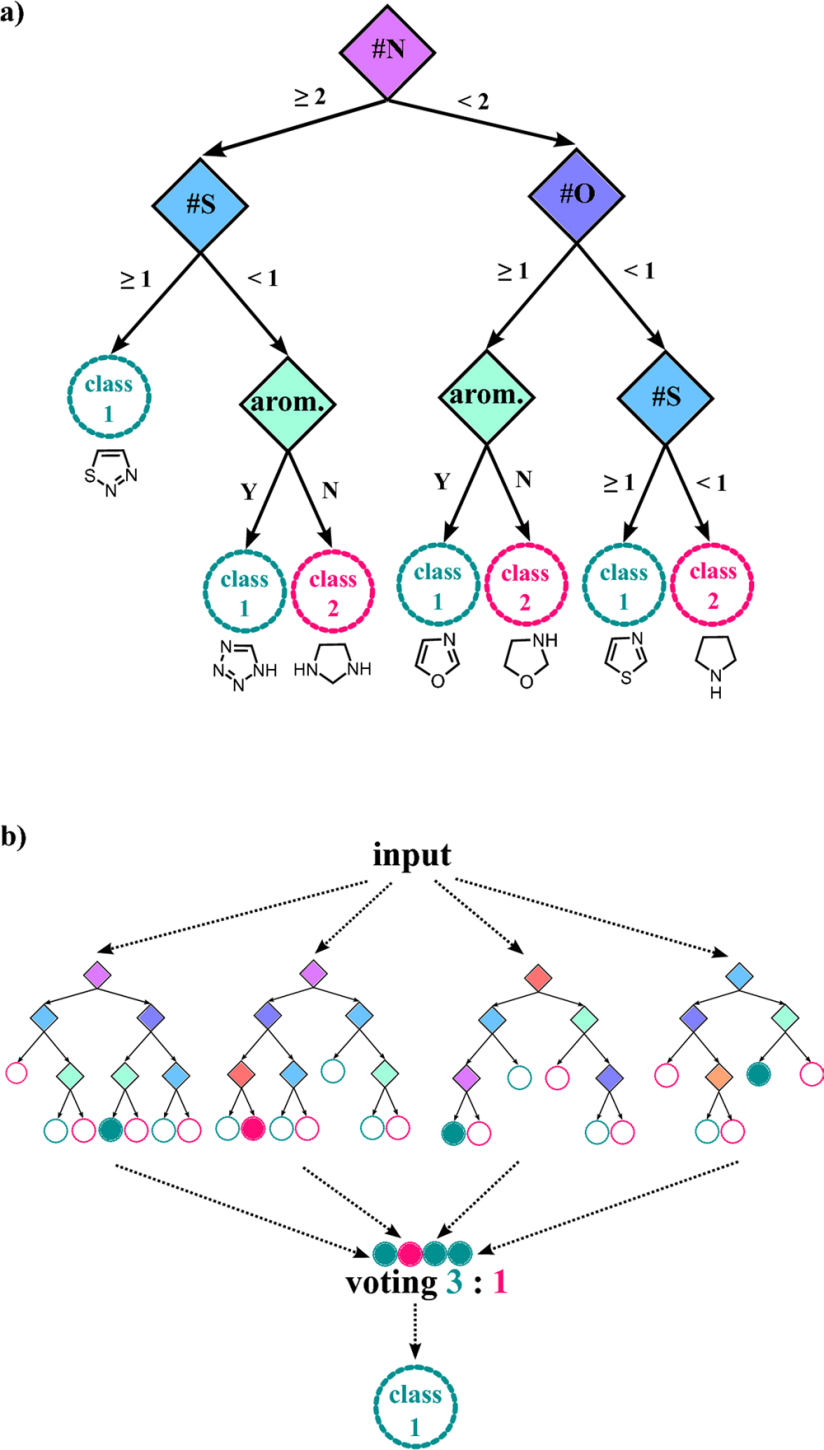
Decision trees and random-forest classifiers. (**a**) An example of a decision tree with simple chemical features classifying input molecules. Traversing the tree top-down, each diamond-shaped node assigns a molecule to a branch depending on its particular chemical feature of interest. For example, oxazole molecule is first classified as having less than two nitrogen atoms (criterion at the purple decision node), then as having at least one oxygen atom (criterion at the dark-blue node), and then as having an aromatic ring (criterion at the light-green node). When sets of molecules are analyzed by such a tree, they are ultimately categorized into two classes – ‘class 1’ corresponding to azoles, and ‘class 2’ corresponding to azolidines. (**b**) Since the trees are relatively small (i.e., have only few decision nodes/layers) classification accuracy for each individual tree can be poor. However, when large numbers of small trees with different features (the so-called Random Forest) are constructed, and each provides its own classification/“vote,” majority vote across all trees enhances classification accuracy. For details of this algorithm please see ref. 18.

Any machine learning necessitates a collection of examples which in the so-called supervised ML methods serve as the training set – in our case, the set of published reactions characterized by certain features/attributes and outcomes. In the so-called decision trees that gave the best results in our study, one splits the dataset into branches corresponding to the presence of certain features until reaching subsets that contain highly homogeneous entries (see example in Fig. 2). The decision trees have been known for a long time but they suffer from high variance of their predictions – a much more precise method is the so-called Random Forest, RF^30, 31^, approach in which the training set is split into subsets, and each is trained on its own decision tree (Fig. 2b). The results are then averaged over the trees decreasing the variance and increasing the accuracy of prediction against data in the test set (i.e., not in the original training set). All results discussed below were based on RF algorithms performed with four-fold cross-validation scheme whereby the entire reaction set is divided into four equal parts, and various combinations of three of these parts are used as training sets (with the remaining, fourth part being a test set); the results are then averaged over these combinations.

## Results

**Figure 3:**
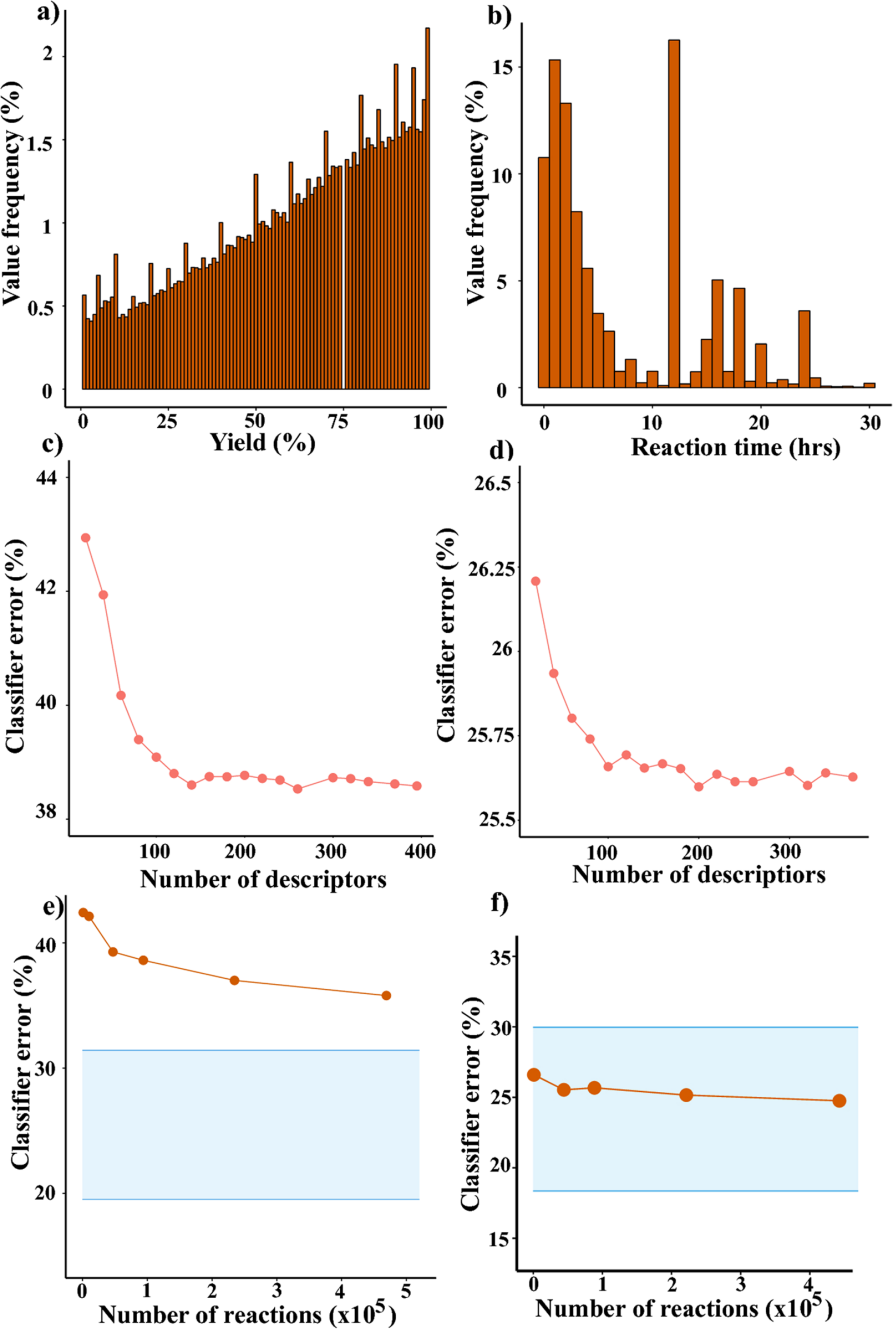
Yield and time predictions based on molecular descriptors. Histograms with distribution of (**a**) yields (**b**) reaction times. Classifier errors plotted as a function of the number of RDKit descriptors for (**c**) yields and (**d**) reaction times. The errors stabilize above c.a. 100 descriptors. Similar analysis (i.e., with classifiers built on descriptors) for different sizes of the reaction sets evidences that errors (both for yields (**e**) and (**f**) times) do not significantly decrease for larger datasets. Shaded, blue regions in panels (**e**) and (**f**) demarcate lower and upper estimates for the Bayes classifier error (i.e. the best classifier that can be used to discriminate between these datasets, see SI for theoretical details).

With these methods, we wished to perform two binary classifications – namely, whether reaction yields and times could be predicted as being above or below certain threshold values. The specific thresholds were 65% for yield (chosen as a median of all yields in the reaction set; see Fig. 3a for the distribution of yields) and 12 hrs for times (chosen as a boundary between reactions with well specified times and those “left overnight” as often conveniently reported; see Fig. 3b for the distribution of reaction times). We emphasize that (i) the results did not differ significantly for other threshold values and (ii) were only worse when more than two outcomes were considered (e.g., low, moderate and high yield classes) or when regression models were used. For instance, the root mean square error in predicting yields via regression based on RF with four-fold cross-validation was as high as 25% on a yield scale 0–100%.

Figure 3c plots the accuracy of yield classification as a function of the number of RDKit descriptors, *N*_*descr*_, used to train the model. Although the error (red markers) gradually decreases with *N*_*descr*_, it levels off at ca. 37% (i.e., accuracy is, at most, 63%) even when as many as 400 descriptors are used. Figure 3d has a similar plot for the accuracy of reaction-time prediction – in this case, the error remains at ca. 26%. Red markers in Fig. 3e and f plot the error as a function of the size of the reaction set used. As seen, even for 450,000 reactions, the errors are still ca. 35% for yields and ca. 25% for times, with the improvements becoming marginal upon increasing the dataset (especially for reaction times).

**Figure 4:**
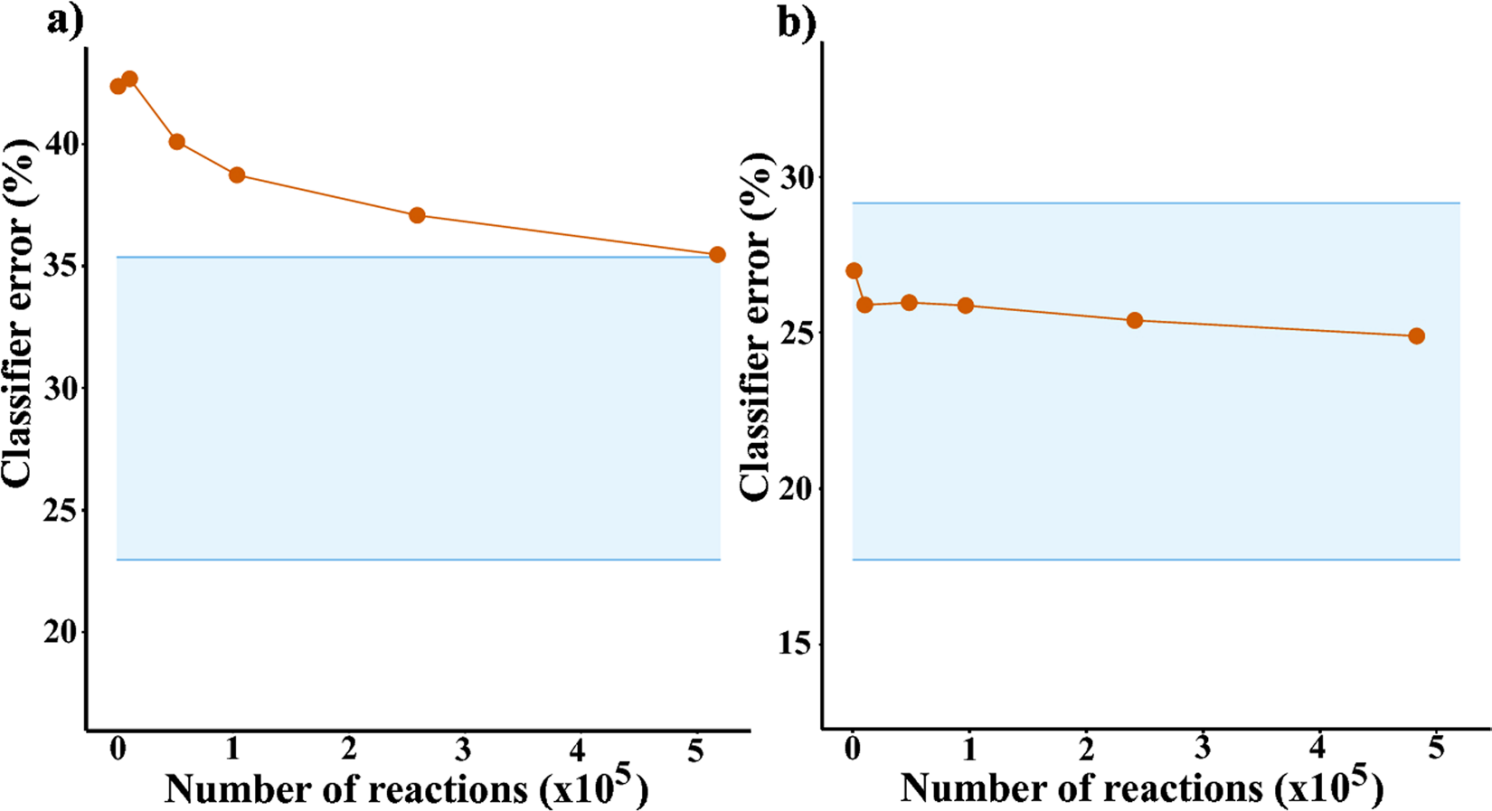
Classifier performance for Random Forests built on reaction fingerprints. Errors – both for (**a**) yields and (**b**) times – do not significantly decrease for large datasets. Blue, shaded regions demarcate lower and upper estimates for the Bayes classifier error.

The results for the analyses based on fingerprints are summarized in Fig. 4. Here, the number of fingerprints is constant (800,000) so the trends are shown as a function of the reaction set size. There is no significant improvement over descriptor-based analyses and the errors are above 35% for yields (Fig. 4a) and 25% for times (Fig. 4b).

It is important to ensure that the above analyses are not biased by having too many, conflicting, and/or irrelevant descriptors. Redundant descriptors (and consequent overfitting) are problematic when the number of descriptors is relatively high compared to the number of observations. In our case, we use 400 common descriptors and/or up to few thousands of chemical-linguistic descriptors, which is small compared to ~0.5 million “observations”/reactions. Also, each of the data points we present in Figs 3 and 4 is for the subset of descriptors that give the highest correlation with either reaction yield or reaction time (e.g., there are multiple ways of choosing 100 descriptors out of the total of 400 – the classification error plotted is for those 100 descriptors that give the highest correlation). This procedure clearly eliminates irrelevant descriptors. Finally, following the methods described in ref. 35, we performed additional tests in which we ran logistic regression with LASSO penalty for a subsample of 90, 000 reactions/”observations. We obtained ~74% accuracy for reaction times and ~60% accuracy for reaction yields, both of which agree with the results we present in the paper.

Additional analyses based on the so-called Gini index^36^ indicated that classifiers’ performance stabilizes when large sets of descriptors are used with the feature-importance score being stable over different algorithm runs (see SI, Figure S4). Principal component analysis, PCA, also confirms the intrinsic complexity of the performed classification task – in particular, data from different classes cannot be separated in the Euclidean space (see SI, Figure S3).

The above results suggest that simply increasing the size of the training set or the number of features/descriptors is unlikely to lead to significantly better results. Yet, one might always speculate that such an improvement were possible with a different classifier structure. However, mathematical methods exist that eliminate such speculation. Specifically, we calculated the so-called Bayes classifier error rate which, in our case, is the probability that a reaction outcome is misclassified by a classifier (e.g., RF) that “knows” *a priori* the true class probabilities (i.e., here, whether the reaction is really low/high yielding or whether its time is long/short) given the molecular or fingerprint predictors used. In other words, the Bayes classification rate estimates the lowest possible error rate for our high-low yield/long-short time classifications and is analogous to the irreducible error. Based on the mathematical considerations detailed in the SI, Section S1, the lower and the upper bounds for the Bayes error can be calculated – these are indicated by the blue lines in Figs 3e,f and 4a,b. As seen, the lower bound for yield-prediction error is ca. 20% and that for the time-prediction error is ca. 17%. This means that while our RF classifiers can be improved slightly, one cannot achieve classification accuracy above ca. 80% which in ML practice is not considered a spectacular result.

**Figure 5:**
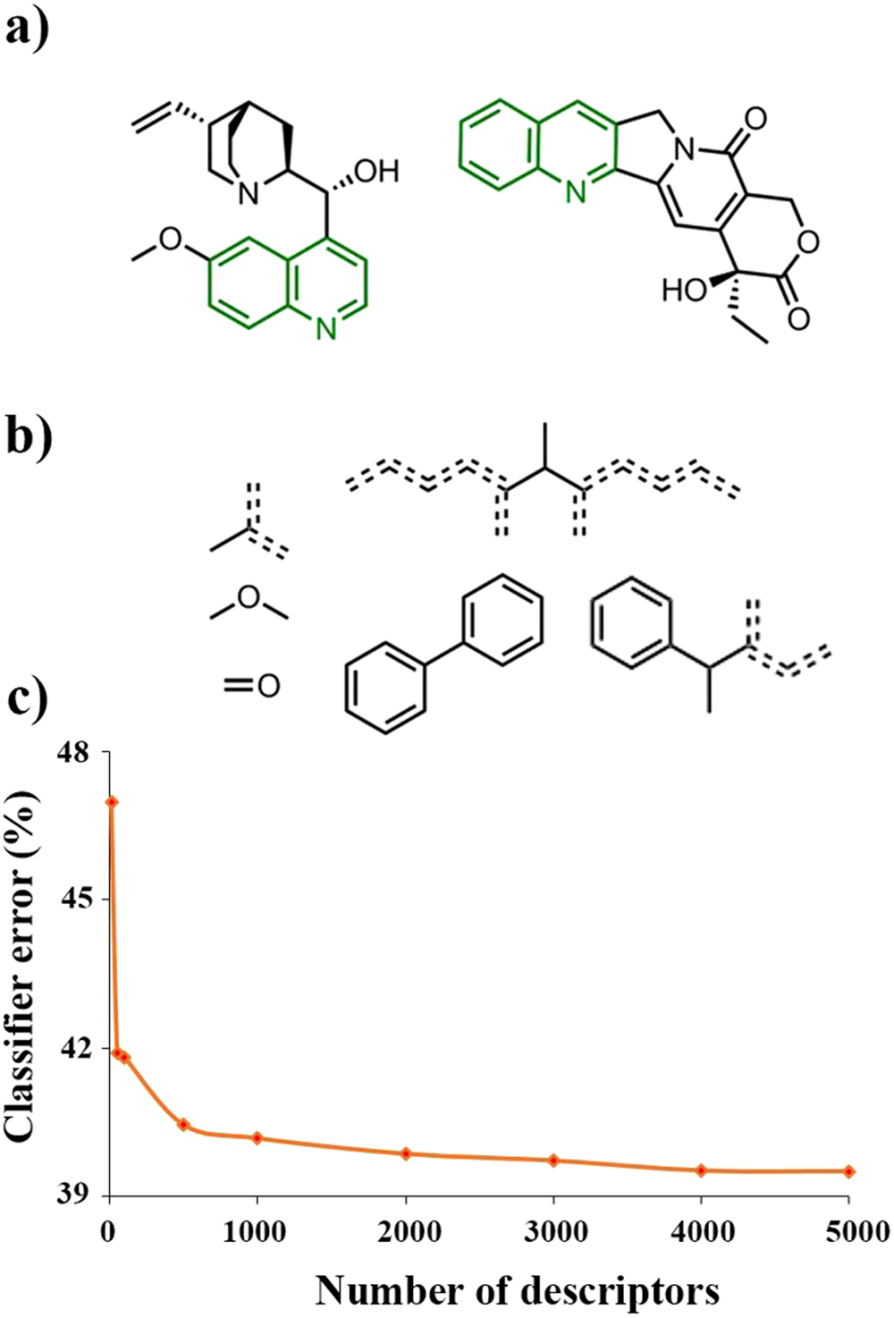
Classification based on chemical-linguistic descriptors. (**a**) An example showing two organic molecules and their maximal common substructure – such substructures computed over millions of molecule-molecule pairs can be used as chemical-linguistic descriptors, CLDs. (**b**) Examples of some smaller and larger CLDs used as descriptors to predict reaction yields and times. Dashed lines denote aromatic bonds. (**c**) Performance of a random forest classifier based on various numbers of CLDs. Even for 5,000 descriptors, the misclassification error is still ca. 40%.

One of the possible reasons for this under-par performance is that the descriptors and/or fingerprints used do not really capture the nuances of molecular structures. Inspection of the descriptors’ list in the SI indicates that they are generally of two types – focusing either on the properties of the entire molecule (electronic properties, topological indices, solubility measures) or on the presence/absence of traditional functional groups. Yet, molecules are often characterized and recognized by the presence of features at an “intermediate” level – for example, when a chemist looks at a steroid, it is not only that presence of a certain number of rings he/she recognizes, but the larger-scale pattern of how these rings are arranged with respect to one another. In one of our recent publications^34^, we defined such characteristic patterns as the maximum common substructures shared by pairs of molecules (Fig. 5a). When millions of such pairs were inspected, they contained tens of thousands of unique substructures ranging from relatively simple fragments to larger motifs implicitly containing in themselves some information about three-dimensional structure (Fig. 5b). Remarkably, when the substructures were then ranked according to their popularity over all molecule pairs, they gave a distribution that was identical with that characterizing the occurrence of common word fragments in the English language – hence, an analogy of the substructures to “chemical words”. Interestingly, these “words” of chemistry were quite informative in identifying most reactive bonds in the molecules to which they belonged (for details, see ref. 34). In the context of our present discussion, we hypothesized that these substructures could also be used as informative chemical-linguistic descriptors, CLDs, carrying in them more information about the molecules than isolated functional groups or simple features such as rings. Accordingly, we tested whether the CLDs could predict reaction yields or times better than the RDKit descriptors. Still, even with as many as 5,000 CLDs, the machine learning methods described earlier showed no improvement. This is illustrated in Fig. 5c which plots the error of yield classification as a function of the number of CLDs used – as seen, the error stabilizes at ca. 40%. As a last attempt, we performed classification with mixed sets of RDKit and CLD descriptors. In that case, the lowest errors we achieved were no better than ~35%.

## Discussion and Outlook

**Figure 6:**
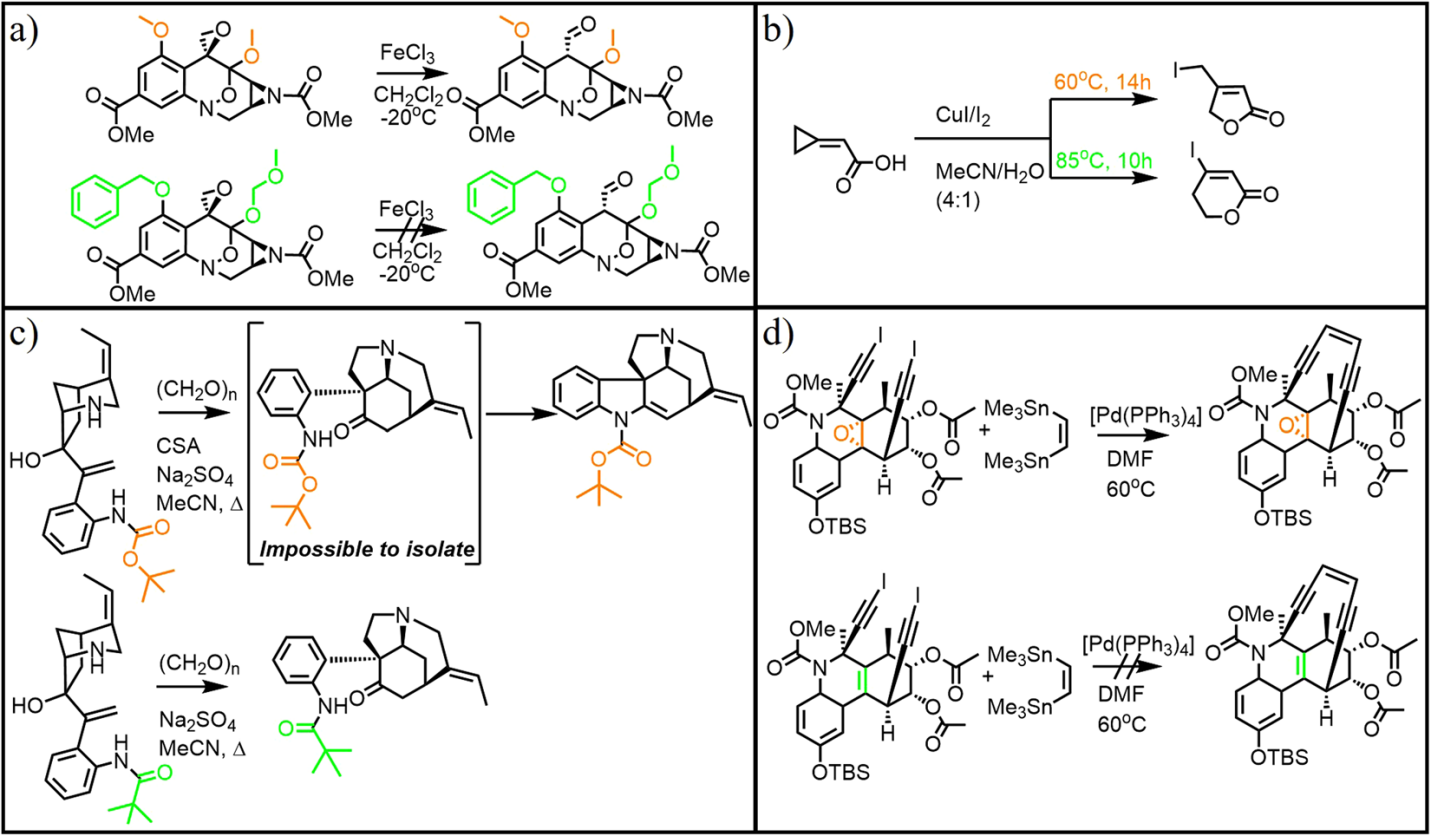
A challenge for Machine Learning: Minor structural changes in starting materials can dramatically influence the reaction outcomes. (**a**) Replacement of two O-protecting groups (orange OMe to green OBn and OMOM) in the intermediate in Danishefsky’s synthesis of (+/−)-FR-900482 changes the lability of ether groups and prohibits rearrangement of an epoxide to an aldehyde^37^. (**b**) Minute changes in temperature alter reaction mechanism and result in different products^38^. (**c**) Small changes in electron density modify reactivity of N-pivaloyl and N-Boc protected anilines. The upper substrate reacts into an intermediate that is impossible to isolate and thus leads to a product that is markedly different than the one obtained from the lower substrate differing in only one atom (oxygen)^39^. (**d**) Presence of the epoxide ring in the tricyclic moiety allows for close proximity of the terminal iodides enabling double Pd-mediated coupling. In contrast, when the epoxide is replaced by a double bond, the iodides are further apart and no cyclization is observed^40^.

The main conclusion from the above analyses is that ML methods are, at least at present, not performing well in predicting the outcomes of organic reactions. One might argue that the results would be improved with some other set of descriptors. On the other hand, we used here virtually all accepted chemoinformatic descriptors and also large numbers of additional chemical-linguistic substructures – it is somewhat hard for us to imagine other sets that would have more chemical content and could offer better predictive power. In fact, we believe the difficulty of the problem is not only in the descriptors but in the form in which organic reactions are presented in organic-chemical literature – namely, that they typically come without full stoichiometry, include some key reagents only as abbreviations, or do not report small by-products. In the thermodynamic model we reported in^22^, all reactions had full stoichiometry, and all bonds broken or made were considered – hence, the model could fully account for reaction enthalpies and performed well with only few hundred free parameters (i.e., less than the number of descriptors we used in current ML models). The problem of incomplete stoichiometry is further compounded by the inherent ambiguity in the reported reaction outcomes (i.e., even for reactions performed by the same team, yields can vary significantly, see ref. 22), calling for a systematic scrutiny and “cleaning-up” of reaction repositories such as Reaxys or SciFinder. In addition, there is a problem of insufficient number of literature examples on which to train the ML models. As we estimated in ref. 23, there are on the order of 10 million known reactions but as many as 20,000–30,000 distinct reaction types, meaning that the statistics for learning are few hundred examples per reaction type, which is generally insufficient for covering the combinations of possible substituents, steric and electronic effects, etc. Last but not least, it is unclear how to account for the cases in which very small alterations in the molecular structures of the reacting molecules can lead to dramatically different reaction outcomes (cf. examples in Fig. 6). We believe that in order to capture such nuances, fundamentally new descriptors should be developed that account not only for the connectivity of molecular graphs, but also for the stereoelectronic properties and three-dimensional conformations of molecules.

## Electronic supplementary material

Supplementary Information
