# Supplementary material for: Predicting the outcomes of organic reactions via machine learning: are current descriptors sufficient?
Source: Sci Rep. 2017 Jun 15;7:3582. doi: 10.1038/s41598-017-02303-0 (PMC5472585; doi:10.1038/s41598-017-02303-0)
Supplement: Supplementary file 1 — Supplementary Information [file 41598_2017_2303_MOESM1_ESM.pdf]

Supplementary Information for Manuscript titled "*Predicting the outcomes of organic reactions via machine learning: are current descriptors sufficient?*" by G. Skoraczyński[1†], P. Dittwald[2†], B. Miasojedow[1], S. Szymkuć[2], E.P. Gajewska[2], B.A. Grzybowski[2,3*], A. Gambin[1*]

[1)] Faculty of Mathematics, Informatics, and Mechanics, University of Warsaw, 02-097 Warsaw, Poland

[2)] Institute of Organic Chemistry, Polish Academy of Sciences, Warsaw, Poland

[3)] Center for Soft and Living Matter of Korea's Institute for Basic Science (IBS), Department of Chemistry, Ulsan National Institute of Science and Technology, Ulsan, South Korea

[†] The authors contributed equally

* e-mail:grzybor72@unist.ac.kr or aniag@mimuw.edu.pl

1. Methods.

This section provides details of the method used to compute the bounds on the accuracy of classifiers of reaction yields and durations.

Classification problem

One of most important tasks of machine learning methods is to predict the value of a certain characteristic, say y (whose evaluation is difficult and computationally expensive), based on vector of features x. For finite possible values of y one then has a classification problem and when y is a real number, such a problem is called regression. In the present work we focus on a binary classification problem, in which for a given chemical reaction we wish to predict whether its yield/duration are, respectively, high-low or long-short. Features used for classification include chemical descriptors, common substructures, information about solvent and temperature, and more.

The binary classification problem is widely described in the ML literature. There are many approaches to this problem including logistic regression, support vector machines (SVM)[1], random forests (RF)[2], *k*-Nearest Neighbors (kNN) and its modifications, etc. As discussed in the main text, RF gave the best performance. Here, our aim was to investigate whether the accuracy of these predictions can, in principle, be improved with some other (hypothetical) classifier architecture. As we show, it is not possible to achieve better accuracy unless some additional knowledge is provided. In order to prove this statement formally, we applied the method proposed recently by V. Berisha et al. in refs [3,4], which allows to estimate the probability of misclassification for the binary Bayes classifier.

Binary Bayes classifier and its accuracy

Let us consider the problem of classifying a feature vector $x \in R^p$, into one of classes $y \in \{0,1\}$. We denote conditional distributions by $f_0(x)$ and $f_1(x)$, respectively, and the prior probability of class 0 by p. The Bayes classifier $\delta(x): R^p \to \{0,1\}$ assigns an observation x to a class with the highest posterior probability and maximizes probability of correct prediction. Although the Bayes classifier is usually unfeasible (since distributions f_0 and f_1 are unknown), its value lies in the fact that other ML techniques cannot achieve better accuracy than the Bayes classifier. Therefore it is reasonable to consider Bayes classifier error rate:

$$e^{Bayes} = P(\delta(x) \neq y)$$

as the measure of difficulty of a problem.

Efficient estimation of the Bayes error rate is complicated. Thus, instead of estimating e^{Bayes} directly, we introduce and then estimate sharp lower and upper bounds on e^{Bayes}. Bounds on e^{Bayes} are based on the following divergence measure $u(\cdot,\cdot)$:

$$u(f_0, f_1) = \int \frac{\left(p \cdot f_0(x) - (1-p) \cdot f_1(x)\right)^2}{p \cdot f_0(x) + (1-p) \cdot f_1(x)} dx$$

Having function u, Bayes error rate e^{Bayes} can be bounded according to Theorem 2 in ref [4]. This theorem states that:

$$\frac{1}{2}-\frac{1}{2}\sqrt{u(f_0,f_1)} \leq e^{Bayes} \leq \frac{1}{2}-\frac{1}{2}u(f_0,f_1)\ .$$

The function $u(f_0,f_1)$ can be estimated by the Friedman-Rafsky (FR) statistic[5]. This statistic entails building a minimum spanning tree (MST) on union of points from different classes and then calculating edges which are incident to vertices from both classes. The number of such edges constitutes a FR statistic. The spanning tree is a subgraph of a given graph, which is a tree (a connected graph with no cycles) incident to all vertices. Minimum spanning tree is a spanning tree which has minimal sum of weights on its edges.

Given the FR statistic, we can estimate function$u(f_0,f_1)$, and further bounds on the Bayes error rate e^{Bayes}. By theorem Theorem 1 from ref [4], we have

$$1-2\frac{FR(X_0,X_1)}{N_0+N_1} \to u(f,g)$$

where $X_0 \in R^{N_0\times dim}, X_1 \in R^{N_1\times dim}$are samples from class 0 and 1, FR is Friedman-Rafsky statistic, and N_0, N_1 are numbers of points in class 0 and 1, respectively.

Bounds on classifier accuracy for yields and times of chemical reactions

Using methods described in the previous section, we estimate the Bayes error rate. To calculate the FR statistic for the set of descriptors, we split them into two subsets, for instance those associated with reactions with high and low yields (e.g., higher or lower than 0.65). As every descriptor is a multidimensional vector, the distances between them are calculated as Euclidean. After splitting points in multidimensional Euclidean space into two classes, we calculated Maximum Spanning Tree (MST) for the union of these two sets using Prim's algorithm[6]. With the MST at hand, we calculated the FR statistic, function u, and then Bayes error rates as described in the previous section. The procedure was repeated several times for different sample sizes with

randomly chosen points. For both reaction yield and duration time, the approximate Bayes error rates stabilize and appear to converge to the true Bayesian prediction error. The results obtained are summarized in Figure S1. The ca. 20% Bayes error rate estimate for our classification problem provides the formal proof that no other classifier can achieve better accuracy given the set of descriptors/fingerprints used to characterize molecules/reactions. For reaction duration dataset, the error's lower bound is smaller but still relatively high (ca. 18%). In Figure S2, analogous results based on the reaction fingerprints are presented. Note that in all cases estimates of upper and lower bounds on the Bayes error rates stabilize for large sample sizes. Thus, our estimates of $e^{Bayes}$are reliable. In addition, the PCA analysis also justifies the intrinsic complexity of the performed classification task, cf. Figure S3. The visualized data from different classes cannot be separated in the Euclidean space.

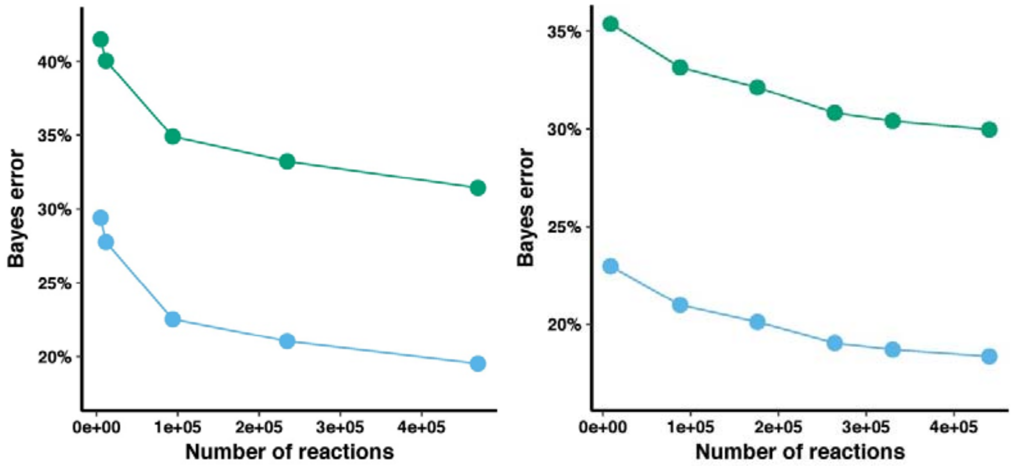

Figure S1. Upper and lower bounds on the Bayes error calculated based on molecular descriptors for different sizes of reaction sets. The left plot is for reaction yields, the right plot is for reaction times.

2. Additional results and analyses.

In addition to the Random Forest classifier, we also tested other machine learning methods. The classifier error for the Extreme Randomized Trees (ERT) was ca. 36% – that is, similar to RF but the classifier worked slower. For the Linear Support Vector Classification (parameter C = 1) the error was about 41%. As discussed in the main text, having constructed the classifiers we performed additional analyses based on the so-called Gini index[7], which indicated that classifiers' performance stabilizes when large sets of descriptors are used with the feature-importance score being stable over different algorithm runs. The results are summarized in Figure S4.

We also attacked the problem using Neural Networks[8,9]. First, we transformed the values of yields into the real line R by logit function ($log\left(\frac{yield}{1-yield}\right)$). Using feed-forward neural networks with single hidden layer and total 270 neurons in all layers, we fitted a linear model with transformed yields as a response variable and with fingerprints as explanatory variables. We used methods and algorithms described in section 8.10 of ref [10]. Finally, for new observation, we assigned a class to which the predicted value of yields belongs. The achieved accuracy was ca. 57% for yield prediction and ca. 74% for duration prediction, which is consistent with the performance of other classifiers.

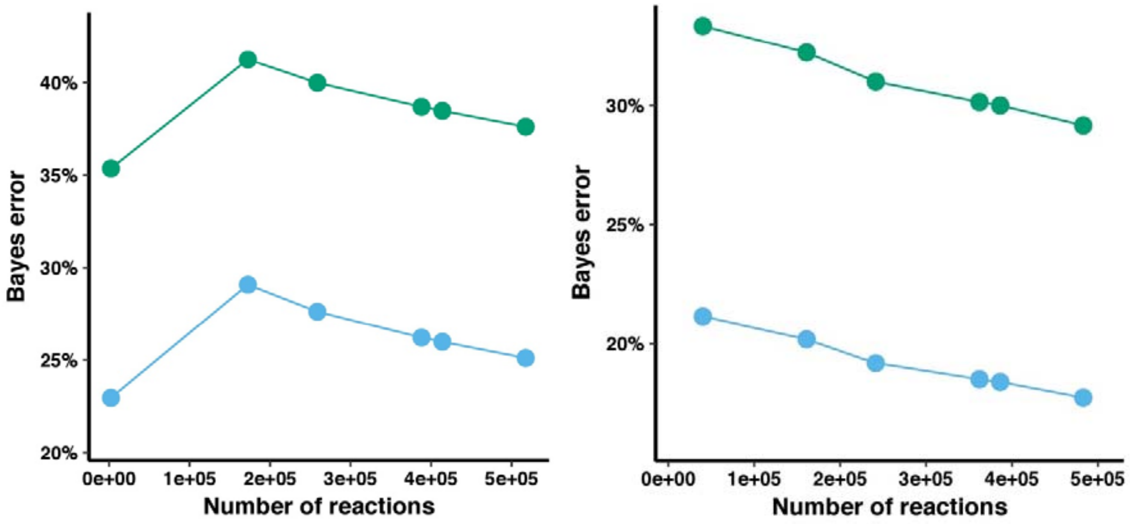

Figure S2. Upper and lower bounds on the Bayes error calculated based on reaction fingerprints for different sizes of reaction sets. The left plot is for reaction yields, the right plot is for reaction times. Note: The smallest error for the smallest number of reactions (in the left portion of the figure, for reaction yields) means that the number of data points was not sufficient to ensure good quality of the Bayes error estimation via the asymptotic theory of the Friedman-Rafsky statistics.

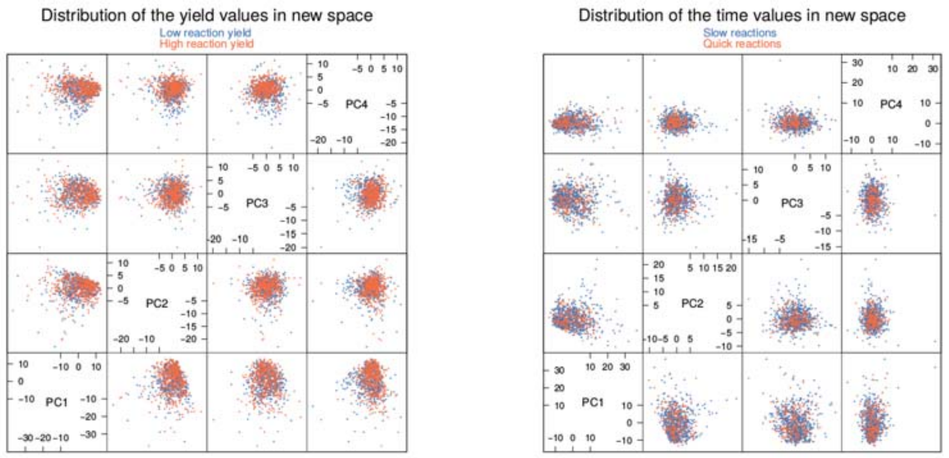

Figure S3. The Principal Component Analysis for reaction yield and duration datasets. Projections into 4 most significant components (explaining more than 50% of the variance) do not reveal any pattern.

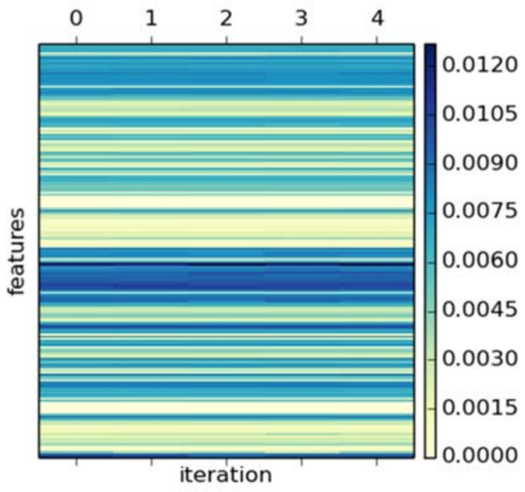

Figure S4. Gini index (GI) of chemical descriptors indicates the importance of a given feature for the classifier's decision. We observe, that GI does not change much between five independent runs of the Random Forest classifier.

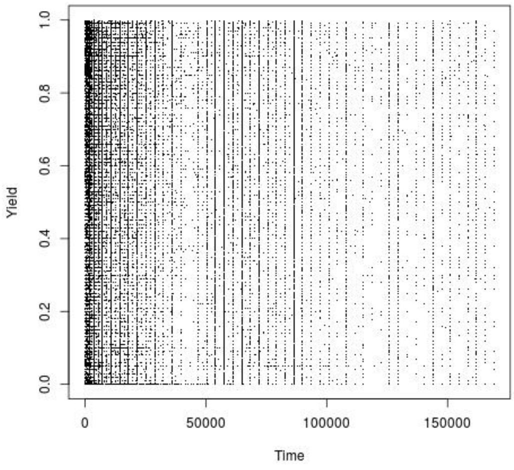

Figure S5. Scatterplot of reaction yields vs. times does not reveal any correlation. The calculated correlation coefficient was 0.06.

Supplementary references.

1. Cortes, C. & Vapnik, V. Support-vector networks. *Mach. Learn.* **20**, 273-297 (1995).
2. Breiman, L. Random forests. *Mach. Learn.* **45**, 5-32 (2001).
3. Berisha, V. & Hero, A.O. Empirical non-parametric estimation of the Fisher information. *IEEE Signal Process. Lett.* **22**, 988-992 (2015).
4. Berisha, V., Wisler, A., Hero, A.O. & Spanias, A. Empirically estimable classification bounds based on a nonparametric divergence measure. *IEEE Trans. Signal Process.* **64**, 580-591 (2016).
5. Friedman, J.H. & Rafsky, L.C. Multivariate generalizations of the Wald-Wolfowitz and Smirnov two-sample tests. *Ann. Stat.* **7**, 697-717 (1979).
6. Cormen, T.H., Leiserson C.E., Rivest, R.L. & Stein, C. *Introduction to algorithms* 2nd ed. (MIT Press and McGraw-Hill, 2001).

7. Menze, B.H. *et al.* A comparison of random forest and its Gini importance with standard chemometric methods for the feature selection and classification of spectral data. *BMC Bioinformatics* **10**, #213 (2009).
8. Hastie T., Tibshirani, R. & Friedman, J. *The elements of statistical learning*, (Springer, 2009).
9. Haykin, S. *Neural networks: A comprehensive foundation* 2nd ed. (Prentice Hall, 1998).
10. Venables, W.N. & Ripley, B.D. *Modern applied statistics with S* 4th ed. (Springer, 2002).

RDKit descriptors

Table S1. Maximal set of RDKit descriptors (which, by being calculated for both substrates and products results in almost 400 descriptors) considered during classification tasks

Nr	Descriptor
1	MinAbsPartialCharge,
2	The number of radical electrons the molecule has (says nothing about spin state),
3	The average molecular weight of the molecule ignoring hydrogens,
4	MaxAbsEStateIndex,
5	MaxAbsPartialCharge,
6	MaxEStateIndex,
7	MinPartialCharge,
8	The exact molecular weight of the molecule,
9	The average molecular weight of the molecule,
10	The number of valence electrons the molecule has,
11	MinEStateIndex,
12	MinAbsEStateIndex,
13	MaxPartialCharge,
14	Calculate Balaban's J value for a molecule,
15	A topological index meant to quantify "complexity" of molecules.,
16	From equations (1),(9) and (10) of Rev. Comp. Chem. vol 2, 367-422, (1991),
17	Chi0n,
18	Chi0v,
19	From equations (1),(11) and (12) of Rev. Comp. Chem. vol 2, 367-422, (1991),
20	Chi1n,
21	Chi1v,
22	Chi2n,
23	Chi2v,
24	Chi3n,
25	Chi3v,

26	Chi4n,
27	Chi4v,
28	HallKierAlpha,
29	This returns the information content of the coefficients of the characteristic polynomial of the adjacency matrix of a hydrogen-suppressed graph of a molecule.,
30	Kappa1,
31	Kappa2,
32	Kappa3,
33	LabuteASA,
34	MOE Charge VSA Descriptor 1 (-inf < x < -0.30),
35	MOE Charge VSA Descriptor 10 (0.10 <= x < 0.15),
36	MOE Charge VSA Descriptor 11 (0.15 <= x < 0.20),
37	MOE Charge VSA Descriptor 12 (0.20 <= x < 0.25),
38	MOE Charge VSA Descriptor 13 (0.25 <= x < 0.30),
39	MOE Charge VSA Descriptor 14 (0.30 <= x < inf),
40	MOE Charge VSA Descriptor 2 (-0.30 <= x < -0.25),
41	MOE Charge VSA Descriptor 3 (-0.25 <= x < -0.20),
42	MOE Charge VSA Descriptor 4 (-0.20 <= x < -0.15),
43	MOE Charge VSA Descriptor 5 (-0.15 <= x < -0.10),
44	MOE Charge VSA Descriptor 6 (-0.10 <= x < -0.05),
45	MOE Charge VSA Descriptor 7 (-0.05 <= x < 0.00),
46	MOE Charge VSA Descriptor 8 (0.00 <= x < 0.05),
47	MOE Charge VSA Descriptor 9 (0.05 <= x < 0.10),
48	MOE MR VSA Descriptor 1 (-inf < x < 1.29),
49	MOE MR VSA Descriptor 10 (4.00 <= x < inf),
50	MOE MR VSA Descriptor 2 (1.29 <= x < 1.82),
51	MOE MR VSA Descriptor 3 (1.82 <= x < 2.24),
52	MOE MR VSA Descriptor 4 (2.24 <= x < 2.45),
53	MOE MR VSA Descriptor 5 (2.45 <= x < 2.75),
54	MOE MR VSA Descriptor 6 (2.75 <= x < 3.05),
55	MOE MR VSA Descriptor 7 (3.05 <= x < 3.63),
56	MOE MR VSA Descriptor 8 (3.63 <= x < 3.80),
57	MOE MR VSA Descriptor 9 (3.80 <= x < 4.00),
58	MOE logP VSA Descriptor 1 (-inf < x < -0.40),

59	MOE logP VSA Descriptor 10 (0.40 <= x < 0.50),
60	MOE logP VSA Descriptor 11 (0.50 <= x < 0.60),
61	MOE logP VSA Descriptor 12 (0.60 <= x < inf),
62	MOE logP VSA Descriptor 2 (-0.40 <= x < -0.20),
63	MOE logP VSA Descriptor 3 (-0.20 <= x < 0.00),
64	MOE logP VSA Descriptor 4 (0.00 <= x < 0.10),
65	MOE logP VSA Descriptor 5 (0.10 <= x < 0.15),
66	MOE logP VSA Descriptor 6 (0.15 <= x < 0.20),
67	MOE logP VSA Descriptor 7 (0.20 <= x < 0.25),
68	MOE logP VSA Descriptor 8 (0.25 <= x < 0.30),
69	MOE logP VSA Descriptor 9 (0.30 <= x < 0.40),
70	TPSA,
71	EState VSA Descriptor 1 (-inf < x < -0.39),
72	EState VSA Descriptor 10 (9.17 <= x < 15.00),
73	EState VSA Descriptor 11 (15.00 <= x < inf),
74	EState VSA Descriptor 2 (-0.39 <= x < 0.29),
75	EState VSA Descriptor 3 (0.29 <= x < 0.72),
76	EState VSA Descriptor 4 (0.72 <= x < 1.17),
77	EState VSA Descriptor 5 (1.17 <= x < 1.54),
78	EState VSA Descriptor 6 (1.54 <= x < 1.81),
79	EState VSA Descriptor 7 (1.81 <= x < 2.05),
80	EState VSA Descriptor 8 (2.05 <= x < 4.69),
81	EState VSA Descriptor 9 (4.69 <= x < 9.17),
82	VSA EState Descriptor 1 (-inf < x < 4.78),
83	VSA EState Descriptor 10 (11.00 <= x < inf),
84	VSA EState Descriptor 2 (4.78 <= x < 5.00),
85	VSA EState Descriptor 3 (5.00 <= x < 5.41),
86	VSA EState Descriptor 4 (5.41 <= x < 5.74),
87	VSA EState Descriptor 5 (5.74 <= x < 6.00),
88	VSA EState Descriptor 6 (6.00 <= x < 6.07),
89	VSA EState Descriptor 7 (6.07 <= x < 6.45),
90	VSA EState Descriptor 8 (6.45 <= x < 7.00),
91	VSA EState Descriptor 9 (7.00 <= x < 11.00),
92	CalcFractionCSP3((Mol)mol) -> float : returns the fraction of C atoms that are SP3 hybridized,

93	Number of heavy atoms a molecule.,
94	Number of NHs or OHs,
95	Number of Nitrogens and Oxygens,
96	CalcNumAliphaticCarbocycles((Mol)mol) -> int : returns the number of aliphatic (containing at least one non-aromatic bond) carbocycles for a molecule,
97	CalcNumAliphaticHeterocycles((Mol)mol) -> int : returns the number of aliphatic (containing at least one non-aromatic bond) heterocycles for a molecule,
98	CalcNumAliphaticRings((Mol)mol) -> int : returns the number of aliphatic (containing at least one non-aromatic bond) rings for a molecule,
99	CalcNumAromaticCarbocycles((Mol)mol) -> int : returns the number of aromatic carbocycles for a molecule,
100	CalcNumAromaticHeterocycles((Mol)mol) -> int : returns the number of aromatic heterocycles for a molecule,
101	CalcNumAromaticRings((Mol)mol) -> int : returns the number of aromatic rings for a molecule,
102	Number of Hydrogen Bond Acceptors,
103	Number of Hydrogen Bond Donors,
104	Number of Heteroatoms,
105	Number of Rotatable Bonds,
106	CalcNumSaturatedCarbocycles((Mol)mol) -> int : returns the number of saturated carbocycles for a molecule,
107	CalcNumSaturatedHeterocycles((Mol)mol) -> int : returns the number of saturated heterocycles for a molecule,
108	CalcNumSaturatedRings((Mol)mol) -> int : returns the number of saturated rings for a molecule,
109	RingCount,
110	Wildman-Crippen LogP value,
111	Wildman-Crippen MR value,
112	Number of aliphatic carboxylic acids,
113	Number of aliphatic hydroxyl groups,
114	Number of aliphatic hydroxyl groups excluding tert-OH,
115	Number of N functional groups attached to aromatics,
116	Number of Aromatic carboxylic acide,
117	Number of aromatic nitrogens,

118	Number of aromatic amines,
119	Number of aromatic hydroxyl groups,
120	Number of carboxylic acids,
121	Number of carboxylic acids,
122	Number of carbonyl O,
123	Number of carbonyl O, excluding COOH,
124	Number of thiocarbonyl,
125	Number of C(OH)CCN-Ctert-alkyl or C(OH)CCNcyclic,
126	Number of Imines,
127	Number of Tertiary amines,
128	Number of Secondary amines,
129	Number of Primary amines,
130	Number of hydroxylamine groups,
131	Number of XCCNR groups,
132	Number of tert-alicyclic amines (no heteroatoms, not quinine-like bridged N),
133	Number of H-pyrrole nitrogens,
134	Number of thiol groups,
135	Number of aldehydes,
136	Number of alkyl carbamates (subject to hydrolysis),
137	Number of alkyl halides,
138	Number of allylic oxidation sites excluding steroid dienone,
139	Number of amides,
140	Number of amidine groups,
141	Number of anilines,
142	Number of aryl methyl sites for hydroxylation,
143	Number of azide groups,
144	Number of azo groups,
145	Number of barbiturate groups,
146	Number of benzene rings,
147	Number of benzodiazepines with no additional fused rings,
148	Bicyclic,
149	Number of diazo groups,
150	Number of dihydropyridines,
151	Number of epoxide rings,

152	Number of esters,
153	Number of ether oxygens (including phenoxy),
154	Number of furan rings,
155	Number of guanidine groups,
156	Number of halogens,
157	Number of hydrazine groups,
158	Number of hydrazone groups,
159	Number of imidazole rings,
160	Number of imide groups,
161	Number of isocyanates,
162	Number of isothiocyanates,
163	Number of ketones,
164	Number of ketones excluding diaryl, a,b-unsat. dienones, heteroatom on Calpha,
165	Number of beta lactams,
166	Number of cyclic esters (lactones),
167	Number of methoxy groups -OCH3,
168	Number of morpholine rings,
169	Number of nitriles,
170	Number of nitro groups,
171	Number of nitro benzene ring substituents,
172	Number of non-ortho nitro benzene ring substituents,
173	Number of nitroso groups, excluding NO2,
174	Number of oxazole rings,
175	Number of oxime groups,
176	Number of para-hydroxylation sites,
177	Number of phenols,
178	Number of phenolic OH excluding ortho intramolecular Hbond substituents,
179	Number of phosphoric acid groups,
180	Number of phosphoric ester groups,
181	Number of piperdine rings,
182	Number of piperzine rings,
183	Number of primary amides,
184	Number of primary sulfonamides,

185	Number of pyridine rings,
186	Number of quarternary nitrogens,
187	Number of thioether,
188	Number of sulfonamides,
189	Number of sulfone groups,
190	Number of terminal acetylenes,
191	Number of tetrazole rings,
192	Number of thiazole rings,
193	Number of thiocyanates,
194	Number of thiophene rings,
195	Number of unbranched alkanes of at least 4 members (excludes halogenated alkanes),
196	Number of urea groups
